# Cross Border Comparison of MRSA Bacteraemia between The Netherlands and North Rhine-Westphalia (Germany): A Cross-Sectional Study

**DOI:** 10.1371/journal.pone.0042787

**Published:** 2012-08-03

**Authors:** Brigitte A. G. L. van Cleef, Jan A. J. W. Kluytmans, Birgit H. B. van Benthem, Anja Haenen, Jos Monen, Inka Daniels-Haardt, Annette Jurke, Alexander W. Friedrich

**Affiliations:** 1 Centre for Infectious Disease Control Netherlands, RIVM National Institute for Public Health and The Environment, Bilthoven, The Netherlands; 2 Department of Medical Microbiology and Infection Prevention, VU University Medical Centre, Amsterdam, The Netherlands; 3 Laboratory for Microbiology and Infection Control, Amphia Hospital, Breda, The Netherlands; 4 North Rhine-Westphalian Center for Health, Münster, Germany; 5 Department of Medical Microbiology, University Medical Center Groningen, University of Groningen, Groningen, The Netherlands; Rockefeller University, United States of America

## Abstract

**Background:**

We describe the impact of methicillin-resistant *Staphylococcus aureus* (MRSA) in two neighbouring regions in Europe with a comparable population size, North Rhine-Westphalia (NRW) in Germany and the Netherlands.

**Methodology/Principal Findings:**

We compared the occurrence of MRSA in blood cultures from surveillance systems. In the Netherlands in 2009, 14 of 1,510 (0.9%) *Staphylococcus aureus* bacteraemia episodes under surveillance were MRSA. Extrapolation using the number of clinical admissions results in a total of 29 MRSA bacteraemia episodes in the Netherlands or 1.8 episodes per 1,000,000 inhabitants. In 2010 in NRW, 1,029 MRSA bacteraemias were reported, resulting in 57.6 episodes of MRSA bacteraemia per 1,000,000 inhabitants: a 32-fold higher incidence than in the Netherlands.

**Conclusion/Significance:**

Based on an estimated attributable mortality of 15%, the Dutch approach would save approximately 150 lives per year by the prevention of bacteraemia only.

## Introduction

*Staphylococcus aureus (S. aureus)* is a member of the commensal flora of the skin and mucous membranes. It is also known to cause serious infections, mostly in hospitalized patients, especially those undergoing dialysis or surgical procedures [1]. Methicillin-resistant *S. aureus* (MRSA) is resistant to beta-lactam antibiotics (penicillins, cephalosporins and carbapenems), and is a public health concern in many countries all over the world.

At first, MRSA emerged in hospitals (hospital-associated: HA-MRSA), but more recently community-associated MRSA (CA-MRSA) emerged and caused illnesses of varying severity outside the hospitals. In 2005, MRSA was first linked to livestock and a genetically distinct group of strains was identified as livestock-associated MRSA (LA-MRSA) [2]. Nowadays, the boundaries between HA-MRSA and CA-MRSA have become less clear and LA-MRSA has entered the arena. MRSA infections are superimposed on the existing infections caused by methicillin-susceptible *S. aureus* (MSSA) and thereby add significantly to the total burden of disease [3].

Within Europe marked inter-country variations exist for the prevalence of MRSA, which can be due to differences in control measures, usage of antimicrobials and numerous other factors [4], [5]. With this study, we describe the large impact of MRSA in two neighbouring and almost equally large populated regions, North Rhine-Westphalia (NRW) in Germany and the Netherlands, by comparing the occurrence of MRSA in blood culture isolates.

## Methods

### Ethics statement

This study made use of available data, retrieved by national or regional surveillance programs. Therefore no additional ethical approval for this study was needed.

### The Netherlands

The study was based on data from the national antibiotic resistance surveillance system in the Netherlands (ISIS-AR) [6]. The 22 participating laboratories serve approximately 50% of all hospital beds. Data from 2009 on *S. aureus* isolates were extracted and evaluated for material of origin; blood isolates were used, as these are objective markers of invasive infections. Only the first blood isolate per patient was included.

In order to compare data from the Netherlands with NRW, extrapolation of MRSA blood cultures from ISIS-AR to the whole of the Netherlands was performed using the proportion of clinical admissions, which were extracted from mandatory annexes from annual reports on 2009 (Available: www.jaarverslagenzorg.nl. Accessed 2012 Feb 6.), excluding one-day and psychiatric admissions. The annexes were only mandatory in 2009, therefore this year was used to compare data from the Netherlands with NRW. Clinical admissions were added up and compared to the national number of clinical admissions in 2009 (Statistics Netherlands. Available: www.cbs.nl. Accessed 2012 Feb 6.), excluding the same two categories.

In order to show the number of MRSA bacteraemia episodes per 1,000,000 inhabitants, population numbers of the Netherlands were derived from Statistics Netherlands (Available: www.cbs.nl. Accessed 2012 Feb 6.). Extrapolation was done under the assumption that the relation between clinical admissions and population density is the same for laboratories participating in the study and for those who do not.

### North Rhine-Westphalia (Germany)

Data from NRW in 2010 were collected from the mandatory reporting of MRSA blood culture isolates from laboratories (State Institute for Health and Work of the state North Rhine-Westphalia. Available: www.liga.nrw.de. Accessed 2011 Nov 9.), which comprises data from all 404 hospitals in NRW. The year 2010 was the first complete year, and this year was used to compare data from NRW with the Netherlands.

The population number of NRW in 2010 was derived from North Rhine-Westphalian company for Information and Technics (Available: www.it.nrw.de. Accessed 2011 Nov 9.).

## Results

### The Netherlands

Data from 2009 from the 22 participating labs in ISIS-AR resulted in 1,512 episodes of *S. aureus* bacteraemia. Of the 1,510 bacteraemia episodes with resistance information, 14 were methicillin resistant (0.9%). Of the patients with MRSA bacteraemias, 79% were male and 71% were older than 65 or younger than two years of age. The median age was 69 years (interquartile range 56–74).

The sum of clinical admissions from hospitals belonging to the participating laboratories in ISIS-AR in 2009 was 903,623, which is 48% of the total of 1,899,000 admissions in the Netherlands. Extrapolating the 14 episodes of MRSA bacteraemia in 2009 to the whole of the Netherlands using the proportion of clinical admissions, there would have been 14*(1,899,000/903,623) = 29.4 episodes of MRSA bacteraemia in 2009 in the Netherlands in total. This can be recalculated into an incidence of 29.4/16,485,787 = 1.8 MRSA bacteraemic episodes per 1,000,000 inhabitants in 2009.

### North Rhine-Westphalia (Germany)

**Figure 1:**
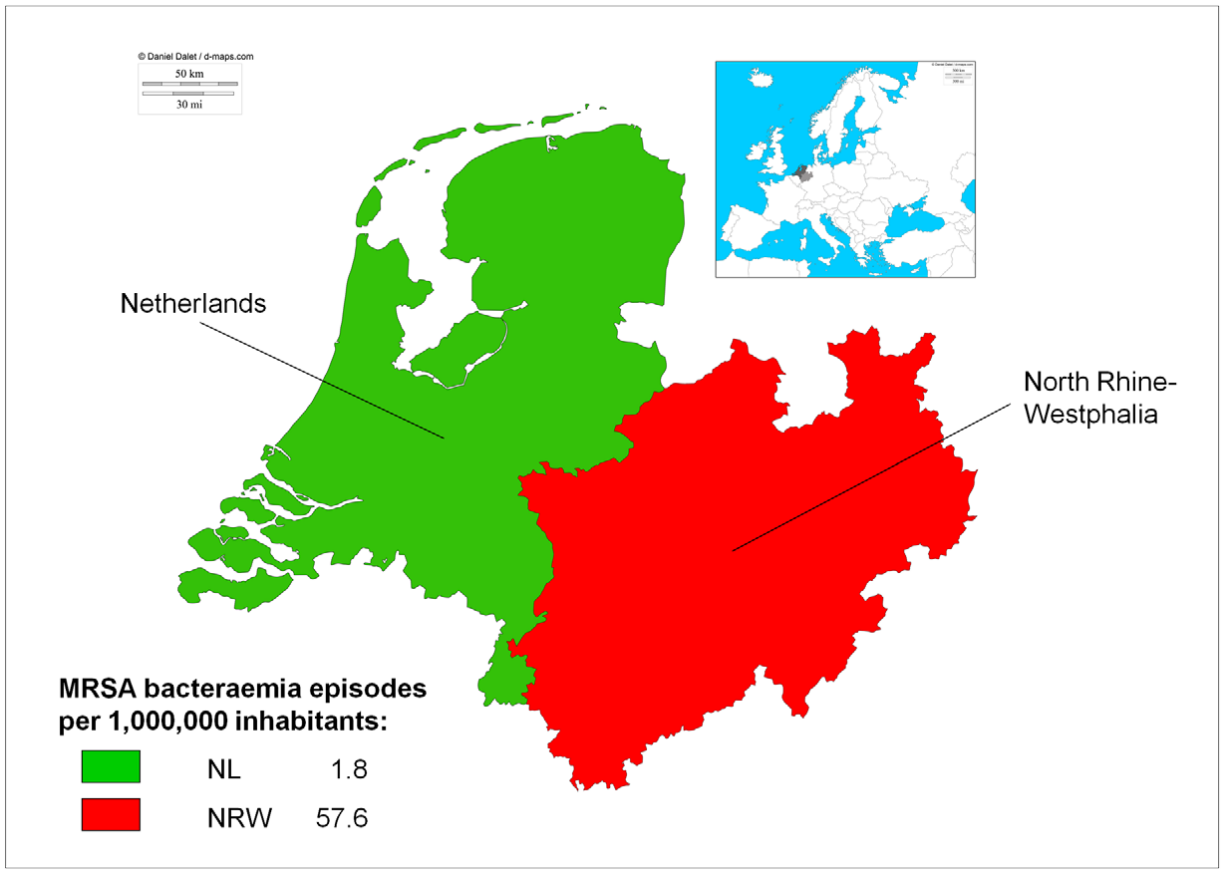
Map of the Netherlands (NL) and North Rhine-Westphalia (NRW). MRSA bacteraemia episodes per 1,000,000 inhabitants for the years 2009 (NL) and 2010 (NRW). A large difference in MRSA bacteraemia episodes was found in this study. Inlay: position of NL and NRW in Europe. Copyright d-maps.com (used maps: paybas22, rhenanienord38, europemax09).

Data from the mandatory reporting of MRSA blood culture isolates from 01 January 2010 to 31 December 2010 comprising all 404 hospitals in NRW resulted in 1,029 episodes of MRSA bacteraemia. This can be recalculated into an incidence of 1,029/17,872,763 = 57.6 MRSA bacteraemias per 1,000,000 inhabitants of NRW, which is 32-fold higher than in the Netherlands (57.6/1.8 = 32.0, Fisher exact p<0.0001, Figure 1). Of the patients with MRSA bacteraemia, 61% were male and 74% were older than 65 or younger than two years of age. The median age was 73 years (interquartile range 65–80).

## Discussion

This study found a large difference in the incidence of MRSA bacteraemias between the region of North Rhine-Westphalia in Germany and the Netherlands. NRW had approximately 1,000 episodes of MRSA bacteraemia more in 2010 than the Netherlands in 2009 (1,029−29.4 = 999), which resulted in a 32-fold higher incidence of MRSA per 1,000,000 inhabitants. The best explanations for this difference in incidence of MRSA bacteraemia in these two adjoining regions with comparable inhabitant numbers are differences in healthcare structure and differences in the implementation of an MRSA control strategy.

### Healthcare structure differences

The differences in healthcare structure can be illustrated with several examples. First, there are grossly 3 times more hospitals in NRW (413 compared to 115 in 2009 in NRW and the Netherlands, respectively), and about twice as much hospital beds (6.8 and 3.3 per 1,000 inhabitants in 2009 in NRW and the Netherlands, respectively) (Statistics Netherlands. Available: www.cbs.nl. Accessed 2012 Feb 6, State Information and Techniques North Rhine-Westphalia. Available: www.it.nrw.de. Accessed 2011 Nov 9, EUROSTAT. Available: http://epp.eurostat.ec.europa.eu/portal/page/portal/eurostat/home/. Accessed 2012 Feb 6.).

Second, in NRW 2.3-fold more patients are admitted to the hospital compared to the Netherlands, while the length of stay is comparable (EUROSTAT. Available: http://epp.eurostat.ec.europa.eu/portal/page/portal/eurostat/home/. Accessed 2012 Feb 6.). More frequent (re)admissons in combination with a higher MRSA proportion among *S. aureus* isolates (26.6% for NRW [7] versus 1.6% for the Netherlands, this study) may lead to a higher introduction rate of MRSA in hospitals in NRW, however this does probably not fully explain the large 32-fold difference in MRSA bacteraemia episodes in this study.

Third, in Germany there are mainly large commercial laboratories that serve large areas, few hospitals have clinical microbiologists available on site. In the Netherlands clinical microbiology laboratories including microbiologists are present in almost every hospital. They are actively involved in the infection control strategy. Recently, in Germany a new law was accepted that made the presence of microbiologists in hospitals mandatory, which might lead to improvements in infection control. It seems that the implementation of an MRSA control policy is more effective in a healthcare structure like the Netherlands.

### MRSA control strategy differences

The ‘search and destroy’ (S&D) strategy in the Netherlands consists of active screening and pre-emptive isolation of persons admitted to foreign hospitals in the previous two months, persons previously positive for MRSA, and persons in contact with live pigs or veal calves (for complete national guidelines see www.wip.nl). Individuals that carry MRSA are actively decolonized with the exception of those who have livestock contact [8]. When unexpected cases are found, active contact tracing is performed.

In Germany there are recommendations actually more strict than the Dutch strategy, with an extra risk group of persons admitted to local hospitals in the last 12 months (for complete German guidelines, see www.rki.de). However, up to now a complete implementation in Germany has only been shown in several regional MRSA-networks (www.eursafety.eu, www.mre-net.org), other regions (among which parts of NRW) have not fully implemented the screening and isolation strategy. This is in contrast to the Netherlands, where the S&D strategy is fully implemented in all hospitals and controlled by the independent national health inspectorate.

The effectiveness of screening regimens depends on various factors, such as the number of patients admitted and re-admitted, the patient-health-care worker ratio [9], the existence of and adherence to hygiene protocols to prevent transmission [10], and host susceptibility [4]. Several articles have studied the effect of individual screening interventions (see [11] for an overview), but so far the effectiveness of the total bundle of the S&D strategy has not been tested. The presence of fully implemented proper national guidelines in all healthcare institutions might be the key to MRSA infection control.

### MRSA prevalence, antimicrobial use and population characteristics

Other factors that may contribute to a higher incidence of MRSA bacteraemia are the prevalence of MRSA in the general population, antimicrobial use and population characteristics. Germany has on average 2.5 MRSA cases per 100 inpatients, in contrast to 0.2 MRSA cases per 100 inpatients in the Netherlands [12], [13], [14], [15]. Moreover, as previously stated, of all *S. aureus* blood isolates in NRW in 2009, 26.6% were MRSA [7], compared to 1.6% in the Netherlands (this study).

Additionally, antimicrobial consumption in ambulatory care in NRW is 16.2 defined daily doses (DDD) per 1,000 inhabitants per day, as opposed to 11 DDD in the Netherlands (ESAC country sheets 2008. Available: www.esac.ua.ac.be. Accessed 2012 Feb 6, Germap 2008. Available: www.bvl.bund.de. Accessed 2011 Nov 9.).

Lastly, when looking at population characteristics or cultural factors [4], [5], the gender and age of patients did not differ between the regions (79% and 61% males, Fisher exact p = 0.29, and 71% and 74% persons older than 65 or younger than two years of age, Fisher exact p = 1.00, in the Netherlands and NRW, respectively).

### Study limitations

This study made use of available data, which bares some limitations. First, selection bias may count for part of the results. In the region of NRW all MRSA blood cultures are supplied, as for the Netherlands only about 50% of admissions are covered, leading to the need to extrapolate these data. The missing hospitals are relatively often located in the larger cities. If hospitals in large cities would have more MRSA bacteraemia episodes, for example because of more foreign patients, patients that travel more often or more complicated patients, the results of this study would underestimate the true MRSA bacteraemia prevalence, and thereby overestimate the difference between the regions studied. On the other hand, the participating laboratories are located more often in pig dense areas where MRSA carriage rates are higher than elsewhere in the Netherlands, hypothetically leading to an overestimation of MRSA bacteraemia prevalence and an underestimation of the difference between the studied regions. However, LA-MRSA carriage does not often result in bacteraemias. Regardless of the above, other studies do not indicate that there are large differences in MRSA prevalences in hospitals within the Netherlands [4], [15]. Therefore we assume that our extrapolation is legitimate.

A second limitation of this study is the difference in time periods that were studied (2009 in the Netherlands and 2010 in NRW, for argumentation see Methods). No major legislational changes have been described for both countries, and MRSA prevalences are estimated to be roughly stable over these years [7], [16]. For these reasons we feel that comparison of these two different years is justifiable.

Third, the effects found in this study could be an underestimation since only bacteraemias were counted. If all MRSA-infections were included, the morbidity, mortality and costs saved will probably be more than demonstrated hereunder. So these assessments can be considered a minimal estimate of the benefits.

### Possible effects

The possible impact of the observed differences can be estimated based on several assumptions. MRSA bacteraemia increases the burden of disease as it does not appear to replace the susceptible strains, but comes on top of it [17], [18]. This would mean that the Dutch approach prevents approximately 1,000 MRSA bacteraemia episodes per year, not only possibly resulting in less morbidity, but also in less mortality and less costs. Several studies show that the attributable one-year mortality of MRSA bacteraemia has a lower limit of 15% (Duin Van D, Fraser T, Jain A, Gordon S, Shresta N. Attributable mortality after S. aureus bacteraemia. Oral presentation at the annual scientific meeting of the Society of Healthcare Epidemiology of America 2011, abstract 299. Available: http://shea.confex.com/shea/1/webprogram/Paper4403.html. Accessed 2012 Feb 6.) [18], [19], [20]. Extrapolating these numbers results in a minimum estimate of 1,000*15% = 150 deaths that would not occur in the Netherlands because of MRSA bacteraemia. According to a recent mathematical model it will also save money as MRSA bacteraemia is associated with substantial costs [21].

### Conclusions

In conclusion, we observed a huge difference in the incidence of MRSA bacteraemia episodes in two comparable and neighbouring regions, North Rhine-Westphalia in Germany and the Netherlands. The estimated annual savings for The Netherlands are at least 150 lives. Next to differences in healthcare structure, the active ‘search and destroy’ strategy in the Netherlands appears to be an efficient way to prevent many MRSA infections.

## References

[1] Lowy FD (1998) *Staphylococcus aureus* infections. N Engl J Med 339: 520–532. 9709046 10.1056/NEJM199808203390806

[2] Voss A, Loeffen F, Bakker J, Klaassen C, Wulf M (2005) Methicillin-resistant *Staphylococcus aureus* in pig farming. Emerg Infect Dis 11: 1965–1966. 16485492 10.3201/eid1112.050428 PMC3367632

[3] Köck R, Becker K, Cookson B, van Gemert-Pijnen JE, Harbarth S, et al (2010) Methicillin-resistant *Staphylococcus aureus* (MRSA): burden of disease and control challenges in Europe. Euro Surveill 15: 19688. 20961515 10.2807/ese.15.41.19688-en

[4] Tiemersma EW, Bronzwaer SL, Lyytikainen O, Degener JE, Schrijnemakers P, et al (2004) Methicillin-resistant *Staphylococcus aureus* in Europe, 1999-2002. Emerg Infect Dis 10: 1627–1634. 15498166 10.3201/eid1009.040069 PMC3320277

[5] Harbarth S, Albrich W, Goldmann DA, Huebner J (2001) Control of multiply resistant cocci: do international comparisons help? Lancet Infect Dis 1: 251–261. 11871512 10.1016/S1473-3099(01)00120-7

[6] Sande-Bruinsma van de N, Leverstein-van Hall M, Boel E, Ruijs G, Alblas J, et al (2011) ISIS-AR and ISISweb: a new surveillance system for antimicrobial resistance surveillance. Poster 1302 at 21st European Congress of Clinical Microbiology and Infectious Diseases. Clin Microbiol Infect 17: S108–S668 [cited 106 February 2012]. Available from: http://onlinelibrary.wiley.com/doi/2010.1111/j.1469-0691.2011.03558.x/pdf.

[7] Schweickert B, Noll I, Feig M, Claus H, Krause G, et al (2011) MRSA-surveillance in Germany: data from the Antibiotic Resistance Surveillance System (ARS) and the mandatory surveillance of MRSA in blood. Eur J Clin Microbiol Infect Dis doi: 10.1007/s10096-011-1511-8. 10.1007/s10096-011-1511-8 22210264

[8] Ammerlaan HS, Kluytmans JA, Berkhout H, Buiting A, de Brauwer EI, et al (2011) Eradication of carriage with methicillin-resistant *Staphylococcus aureus*: effectiveness of a national guideline. J Antimicrob Chemother 66: 2409–2417. 21719473 10.1093/jac/dkr243

[9] Clements A, Halton K, Graves N, Pettitt A, Morton A, et al (2008) Overcrowding and understaffing in modern health-care systems: key determinants in meticillin-resistant *Staphylococcus aureus* transmission. Lancet Infect Dis 8: 427–434. 18582835 10.1016/S1473-3099(08)70151-8

[10] Verhoeven F, Hendrix MGR, Friedrich AW, Daniels-Haardt I, Nijland N, et al (2007) A web-based infectious disease communication system to enhance healthcare workers' knowledge, attitude, and risk perception about safe work practices. Technology and Health Care 15: 366–367.

[11] Tacconelli E, Johnson AP (2011) National guidelines for decolonization of methicillin-resistant *Staphylococcus aureus* carriers: the implications of recent experience in the Netherlands. J Antimicrob Chemother 66: 2195–8. 21807740 10.1093/jac/dkr309

[12] Köck R, Brakensiek L, Mellmann A, Kipp F, Henderikx M, et al (2009) Cross-border comparison of the admission prevalence and clonal structure of meticillin-resistant *Staphylococcus aureus* . J Hosp Infect 71: 320–326. 19201056 10.1016/j.jhin.2008.12.001

[13] Woltering R, Hoffmann G, Daniels-Haardt I, Gastmeier P, Chaberny IF (2008) [Prevalence of methicillin-resistant *Staphylococcus aureus* (MRSA) in patients in long-term care in hospitals, rehabilitation centers and nursing homes of a rural district in Germany]. Dtsch Med Wochenschr 133: 999–1003. 18446675 10.1055/s-2008-1075683

[14] Wertheim HF, Vos MC, Boelens HA, Voss A, Vandenbroucke-Grauls CM, et al (2004) Low prevalence of methicillin-resistant *Staphylococcus aureus* (MRSA) at hospital admission in the Netherlands: the value of search and destroy and restrictive antibiotic use. J Hosp Infect 56: 321–325. 15066745 10.1016/j.jhin.2004.01.026

[15] Kaiser AM, Haenen AJ, de Neeling AJ, Vandenbroucke-Grauls CM (2010) Prevalence of methicillin-resistant *Staphylococcus aureus* and risk factors for carriage in Dutch hospitals. Infect Control Hosp Epidemiol 31: 1188–1190. 20868286 10.1086/656744

[16] SWAB (2011) NethMap 2011 - Consumption of antimicrobial agents and antimicrobial resistance among medically important bacteria in the Netherlands. Available: www.swab.nl.

[17] Ammerlaan H, Herwaldt L, Pottinger J, Hanberger H, Lingaas E, et al. (2010) Trends in nosocomial MRSA bacteraemias: addition or replacement? In: Ammerlaan H, editor.The clinical impact of methicillin-resistant *Staphylococcus aureus* on morbidity, mortality, and burden of disease. Enschede, the Netherlands: Gildeprint Drukkerijen. 117–142.

[18] de Kraker ME, Wolkewitz M, Davey PG, Koller W, Berger J, et al (2011) Clinical impact of antimicrobial resistance in European hospitals: excess mortality and length of hospital stay related to methicillin-resistant *Staphylococcus aureus* bloodstream infections. Antimicrob Agents Chemother 55: 1598–1605. 21220533 10.1128/AAC.01157-10 PMC3067153

[19] Blot SI, Vandewoude KH, Hoste EA, Colardyn FA (2002) Outcome and attributable mortality in critically Ill patients with bacteremia involving methicillin-susceptible and methicillin-resistant *Staphylococcus aureus* . Arch Intern Med 162: 2229–2235. 12390067 10.1001/archinte.162.19.2229

[20] Grundmann H, Aanensen DM, van den Wijngaard CC, Spratt BG, Harmsen D, et al (2010) Geographic distribution of *Staphylococcus aureus* causing invasive infections in Europe: a molecular-epidemiological analysis. PLoS Med 7: e1000215 doi:10.1371/journal.pmed.1000215. 20084094 10.1371/journal.pmed.1000215 PMC2796391

[21] Wassenberg MW, de Wit GA, van Hout BA, Bonten MJ (2010) Quantifying cost-effectiveness of controlling nosocomial spread of antibiotic-resistant bacteria: the case of MRSA. PLoS One 5: e11562 doi:10.1371/journal.pone.0011562. 20661278 10.1371/journal.pone.0011562 PMC2905392

